# Watermarking Based on Compressive Sensing for Digital Speech Detection and Recovery †

**DOI:** 10.3390/s18072390

**Published:** 2018-07-23

**Authors:** Wenhuan Lu, Zonglei Chen, Ling Li, Xiaochun Cao, Jianguo Wei, Naixue Xiong, Jian Li, Jianwu Dang

**Affiliations:** 1 School of Computer Software, Tianjin University, Tianjin 300350, China; wenhuan@tju.edu.cn (W.L.); zongleitju@gmail.com (Z.C.); li_ling_503@163.com (L.L.); jianguo@tju.edu.cn (J.W.); lijian158@tfri.com.cn (J.L.); 2 Institute of Information Engineering, Chinese Academy of Sciences, Beijing 100093, China; caoxiaochun@iie.ac.cn; 3 School of Computer Science and Technology, Qinghai Nationalities University, Xining 810007, China; 4 School of Computer Science and Technology, Tianjin University, Tianjin 300350, China; jdang@jaist.ac.jp; 5 School of Information Science, Japan Advanced Institute of Science and Technology, Ishikawa 923-1292, Japan

**Keywords:** digital watermarking, self-recovery, speech detection, discrete cosine transform, compressive sensing

## Abstract

In this paper, a novel imperceptible, fragile and blind watermark scheme is proposed for speech tampering detection and self-recovery. The embedded watermark data for content recovery is calculated from the original discrete cosine transform (DCT) coefficients of host speech. The watermark information is shared in a frames-group instead of stored in one frame. The scheme trades off between the data waste problem and the tampering coincidence problem. When a part of a watermarked speech signal is tampered with, one can accurately localize the tampered area, the watermark data in the area without any modification still can be extracted. Then, a compressive sensing technique is employed to retrieve the coefficients by exploiting the sparseness in the DCT domain. The smaller the tampered the area, the better quality of the recovered signal is. Experimental results show that the watermarked signal is imperceptible, and the recovered signal is intelligible for high tampering rates of up to 47.6%. A deep learning-based enhancement method is also proposed and implemented to increase the SNR of recovered speech signal.

## 1. Introduction

With the development of Internet and communication technology, the utilization of multimedia data is becoming common in our daily life, but multimedia data can be tampered with easily, it may be distorted during spreading through the Internet, and may be manipulated by an adversary. It is important to develop algorithms to protect and recover data. The watermark algorithm is a powerful kind of tool for multimedia authentication and verification, as well as tampering recovery. One advantage of watermark algorithms is that the algorithm can localize the tampering area, and on that basis, a number of watermarking schemes aiming at recovering multimedia data have been developed [1,2]. Many watermark algorithms used for the self-recovery in the image literature have been proposed in [3,4]. However, in this paper, we propose an imperceptible, fragile, blind watermark scheme for digital speech. The fragile watermark algorithm has a great advantage in that it locates the damaged position. This advantage is especially evident in the self-recovery watermark algorithm [5,6]. There have been many achievements in the image field [7,8] and video field [9]. Different from the common watermark technique, the application of our watermark scheme is not the integrity verification, but to protect the signal itself. The signal may face a problem of packet loss or even malicious tampering, making some parts of the signal to be lost or the content changes a lot during transmission in a channel. Our scheme is designed to solve these problems by recovering the tampered signal. In our scheme, before a signal is transmitted, a watermark is embedded. In addition, the recipient can recover the tampered signal from the watermark. The watermark itself is imperceptible, exerts almost no effect on the signal, and during the recovery process, our watermark algorithm can recover the cover signal without other information from the original signal. The method we propose is blind watermarking. In addition, many non-blind watermarking techniques have also been developed [10].

The related research is very common in the digital image protection domain [11,12,13,14]. There are many research works on what coefficients are used in representation. Jiri Fridrich [6] and Xunzhan Zhu [15] employed discrete cosine transform (DCT) coefficients, Gurkan Gur [16] employed a low-resolution version of the original image as the data representing the image and Rafiullah Chamlawi [17] and Hisashi INOUE [18] employed wavelet coefficients. When the data or the coefficients are embedded in the host, one common method is to embed data from one region in another region. However, this kind of algorithm has a problem, in that when both regions of the host and the data represents it are tampered, the recovery work is impossible. One of the previous works [19] discovered and solved this problem in the image domain. In addition, this work called such a problem the “tampering coincidence problem”. Meanwhile, if neither the origin data nor the watermark are tampered with, the watermark is waste. When this happens, the watermark data is not employed efficiently. This phenomenon is called the “watermark-data waste problem”. The watermark-data waste problem is severe in the watermark scheme, which uses two regions of watermark [20,21] to represent one region of the host, though this kind of watermark scheme can solve the tampering coincidence problem effectively. As a trade-off between the two problems, references [22,23] proposed a reference-sharing mechanism, where the coefficients are calculated from many regions, and when embedding, the coefficients are embedded into these regions.

The research on self-recovery watermarks in the speech domain is not as voluminous as that in the image domain. A few schemes [24,25] have been extended to speech data. However, most of the schemes have simply been designed and do not consider the trade-off between the watermark data waste problem and tampering coincidence problem. In this study, we focus on the trade-off of the two problems.

In this paper, we extend a watermark scheme from the image domain [26] to the speech signal domain. To avoid the impact of inverse Fourier transformation on the fragile watermark, we apply the method not to the spectrum, which is more similar to a digital image, but to the time domain of the signal. The scheme avoids both the watermark data waste problem and tampering coincidence problem. The method embeds the watermark into the LSBs of the speech signal [27]. The embedded watermark data derives from discrete cosine transform (DCT) coefficients of the host speech, not from an approximate version of the host; our method therefore makes the number of embedded watermarks lower. The watermark data would be extracted from the frames that are not tampered with and act on the frames that are tampered with. We employ the compressive sensing technique [28,29] to retrieve the DCT coefficients by exploiting their sparseness. The more watermark data we can extract, the better the result is. Our watermarking algorithm is based on audio characteristics. The method can be adapted to audio. Although the signal recovered through the watermark is already intelligible, we also use deep learning methods for speech enhancement to improve the signal-noise ratio of the recovered speech signal. We conduct several experiments to compare the enhancement effects of different neural network architectures.

## 2. Watermarking Embedding Procedure

**Figure 1:**
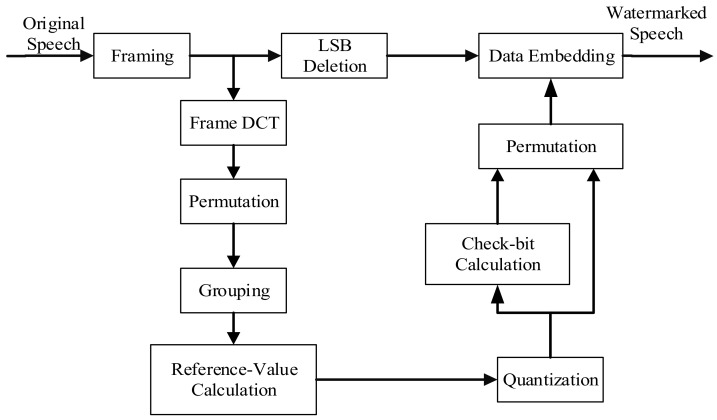
Sketch of watermark embedding procedure.

The watermark algorithm consists of two parts: the watermark embedding part and data recovery part. In this study, we embed the watermark into the LSBs of signal. The reference data are generated from a quantization of a linear representation of original DCT coefficients. Figure 1 shows a sketch of the watermark embedding procedure.

We partition the speech signal into non-overlapping frames, each frame contains N data points. Then, DCT is performed in each frame to yield the DCT coefficients.

(1) $$X(k)=\alpha (k)\sum_{n=0}^{N-1}x(n)\cos[\frac{\pi (2n+1)k}{2N}]\begin{array}{ccc} & & 0\leq k\leq N-1 \end{array}\;$$

where

(2) $$\alpha (k)=\left\{ \begin{array}{l} 1/\sqrt{N},\begin{array}{ccc} & & k=0 \end{array}\\ \sqrt{2/N},\begin{array}{ccc} & & 1\leq k\leq N-1 \end{array} \end{array}\right.\;$$

where x(n) is the original speech signal, X(n) is the DCT coefficients of the original signal.

According to a secret key, pseudo-randomly permute the DCT coefficients in frame units. Assume that each frame group contains m frames. Combine the adjacent m frames into one vector, whose length is n×m where n equal to N, the coefficient-vector is

(3) $$v=\left[C_{i1}^{1},C_{i1}^{2},C_{i1}^{3},...,C_{i1}^{n},C_{i2}^{1},C_{i2}^{2},C_{i2}^{3},...,C_{i2}^{n},C_{i3}^{1},C_{i3}^{2},C_{i3}^{3},...,C_{i3}^{n},...,C_{im}^{1},C_{im}^{2},C_{im}^{3},...,C_{im}^{n}\right]\;$$

And calculate k reference values where k is the number of reference values in one frame-group in the following linear manner.

(4) $$\left[\begin{array}{l} r_{1}\\ r_{2}\\ \vdots \\ r_{k} \end{array}\right]=A\cdot v\;$$

where A is a pseudo-random matrix and the Euclidean norm of each row is one. For generating A, we may first produce a matrix $A_{0}$ sized k×m×n whose elements are derived from a secret key and satisfy an independent identical Gaussian distribution with zero mean. Then, the matrix A can be obtained.

(5) $$A(i,j)=\frac{A_{0}(i,j)}{\sqrt{{\sum_{t=1}^{n\times m}\left[A_{0}(i,t)\right]}^{2}}}\begin{array}{ccc} & 1\leq i\leq k,1\leq j\leq n\times m & \end{array}\;$$

We allocate the k reference value in m frames each group, k/m reference values each frame, and then we quantize the reference values in a uniform manner.

(6) $$\hat{r}=\left\{ \begin{array}{ll} R_{\max}-1, & r\geq f_{R_{\max}}\\ t, & f_{t}\leq r\leq f_{t+1}\\ -t-1, & -f_{t+1}\leq r\leq -f_{t}\\ -R_{\max}, & r\leq -f_{R_{\max}} \end{array}\right.$$

where

(7) $$f(t)=q/R_{\max}\cdot t\;$$

where $R_{\max}$ represents the maximum data after quantification, q is the quantitative parameter. The quantization changes the float reference values into integers to meet the storage constraint. 

We calculate l check-bits for tampered areas localization. We collect the MSBs of one frame, the position of the frame, and the reference values, then import them into a hash function to produce l bits’ hash-bits. Randomly, we generate l bits’ label-bits. For all frames, the label-bits are identical. Then, we calculate the exclusive-or result between the hash-bits and label-bits.

(8) $$c_{i}(j)=h_{i}(j)⊕l_{i}(j),\begin{array}{ccc} & & i=1,2,...,N/n,\begin{array}{cc} & j=1,2,...l \end{array} \end{array}\;$$

where $h_{i}(1),h_{i}(2),...h_{i}(31)$ are the hash-bits, $l_{i}(1),l_{i}(2),...l_{i}(31)$ are the random label-bits, and $c_{i}(1),c_{i}(2),...c_{i}(31)$ are the check-bits. The result is l check-bits. We add the check-bits to the reference values, and regard these bits as our watermark. In our scheme, we set n equal to 64, m equal to 16, and k equal to 368; thus, each frame contains 23 reference values, l equal to 31, $R_{\max}$ equal to 16,384 and q equal to 1500. By quantization, each reference value is converted into an integer within [−16,384, 16,383]. Therefore, each $\hat{r}$ occupies 15 bits, so the number of bits in a frame for reference values is 345. Combine the 345 reference-value-bits, 31 check-bits and eight bits equal to zero. These 384 bits are used to replace the six planes of the frame, producing the watermarked speech. 

## 3. Signal Recovery Procedure

**Figure 2:**
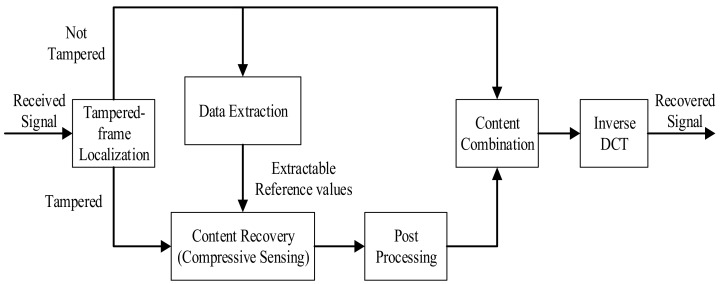
Sketch of watermark recovery procedure.

A watermarked speech signal may be tampered with when transformed via the Internet. Our motivation is to recover the original content. For achieving this aim, we should localize the tampered areas, extract the correct reference values and employ the reference values to calculate the original speech data. For tampered areas localization, we compare the check-bits of every frame group. To recover the original content, we employ the compressive sensing technique to retrieve the DCT coefficients by exploiting their sparseness. Figure 2 shows a sketch of the watermark recovery procedure. The details of each part are as follows.

### 3.1. Tampered Area Localization

The first step of signal recovery is to localize which part of the signal has been tampered with. Dividing the received signal into non-overlapping frames in the same manner as watermark embedding, we then extract the 384 bits from the six LSB-layers, which consist of 345 reference value bits and 31 check-bits. For each frame, we feed the 640 bits in the MSB-layers, the 64 position-bits and the 345 reference bits into the hash function to calculate the 31 hash-bits. Then, we calculate the exclusive-or between the 31 hash-bits and the 31 check-bits.

(9) $$l_{i}(j)=h_{i}(j)⊕c_{i}(j),\begin{array}{ccc} & & i=1,2,...,N/n,\begin{array}{cc} & j=1,2,...,31 \end{array} \end{array}\;$$

where $h_{i}(1),h_{i}(2),...h_{i}(31)$ are the obtained hash-bits, $c_{i}(1),c_{i}(2),...c_{i}(31)$ are the extracted check-bits, and we regard the label-bits $l_{i}(1),l_{i}(2),...l_{i}(31)$ as the result. Comparing the results among all frames, frames whose label-bits are identical to most label-bits are judged as being not tampered. Otherwise, we consider the frame to be tampered. Although a receiver does not know the original label-bits, he can compare the calculated label-bits of all frames. If more than 40% of the frames possess the same result, the receiver can judge them as “reserved” frames and the other frames as “tampered” frames. Clearly, an unmodified frame must be judged as “reserved” as long as the rate of tampered frames is less than 60%. The probability for a frame containing modified content but being falsely judged as “reserved” is $2^{-31}$, which is extremely low, indicating that false judgment is virtually impossible.

### 3.2. Data Recovery

If all frames within the concerned region are not tampered, or all frames are reserved, there is no need to recover. If some frames within the concerned region have been tampered, we would extract (16−z)×23 reference values for recovery, where z is the number of tampered frames. After removing the tampered z frames, Equation (4) is reduced to

(10) $$\left[\begin{array}{l} r(\alpha_{1})\\ r(\alpha_{2})\\ \vdots \\ r(\alpha_{M}) \end{array}\right]=A^{\left(E\right)}\cdot v\;$$

where vector $\alpha_{1},\alpha_{2},...,\alpha_{M}$ are the reference values extracted from reserving frames whose length is M=(16−z)×23, matrix $A^{\left(E\right)}$ is a matrix whose rows are taken from matrix A, corresponding to reserved frames. The vector v could be decomposed into two parts: the DCT coefficients $v_{R}$ from the reserved area, the DCT coefficients $v_{T}$ from tampered area. In addition, our purpose is to find the vector $v_{T}$.

(11) $$\left[\begin{array}{l} r(\alpha_{1})\\ r(\alpha_{2})\\ \vdots \\ r(\alpha_{M}) \end{array}\right]=A^{\left(E,R\right)}\cdot v_{R}+A^{\left(E,T\right)}\cdot v_{T}\;$$

where matrix $A^{\left(E,R\right)}$ is a matrix whose columns are those in matrix $A^{\left(E\right)}$ corresponding to vector $v_{R}$, and matrix $A^{\left(E,T\right)}$ is the counterpart of the vector $v_{T}$. The vector $v_{R}$ could be obtained from the watermarked signal directly.

Deal with the extractable reference values $\alpha_{1},\alpha_{2},...,\alpha_{M}$ to get the original reference-values which are not quantized.

(12) $$r\in \left\{ \begin{array}{l} \left[f_{R_{\max}},+\infty \right),\begin{array}{cccc} & & & \hat{r}=R_{\max}-1 \end{array}\\ \left[f_{\hat{r}},f_{\hat{r}+1}\right),\begin{array}{cccc} & & & 0\leq \hat{r}\leq R_{\max}-2 \end{array}\\ \left[-f_{-\hat{r}},-f_{-\hat{r}-1}\right),\begin{array}{cccc} & & -(R_{\max}-1)\leq \hat{r}\leq -1 & \end{array}\\ \left(-\infty ,-f_{R_{\max}}\right),\begin{array}{cccc} & & & \hat{r}=-R_{\max} \end{array} \end{array}\right.\;$$

Denote

(13) $$r^{\prime }=\left\{ \begin{array}{l} \frac{f_{\hat{r}}+f_{\hat{r}+1}}{2},\begin{array}{ccc} & & 0\leq \hat{r}\leq R_{\max}-1 \end{array}\\ \frac{-f_{-\hat{r}}-f_{-\hat{r}-1}}{2},\begin{array}{ccc} & & -R_{\max}\leq \hat{r}\leq -1 \end{array} \end{array}\right.\;$$

And

(14) $$\left[\begin{array}{l} r^{\prime }(\alpha_{1})\\ r^{\prime }(\alpha_{2})\\ \vdots \\ r^{\prime }(\alpha_{M}) \end{array}\right]\approx A^{\left(E,R\right)}\cdot v_{R}+A^{\left(E,T\right)}\cdot v_{T}\;$$

$\alpha {'}_{1},\alpha {'}_{2},...,\alpha {'}_{M}$ are reference values which are not quantized.

Then

(15) $$\left[\begin{array}{l} S_{1}\\ S_{2}\\ \vdots \\ S_{M} \end{array}\right]=\left[\begin{array}{l} r^{\prime }(\alpha_{1})\\ r^{\prime }(\alpha_{2})\\ \vdots \\ r^{\prime }(\alpha_{M}) \end{array}\right]-A^{\left(E,R\right)}\cdot v_{R}\;$$

And

(16) $$\left[\begin{array}{l} S_{1}\\ S_{2}\\ \vdots \\ S_{M} \end{array}\right]\approx A^{\left(E,T\right)}\cdot v_{T}\;$$

We employ the compressive sensing technique to solve $v_{T}$ by exploiting the sparseness of the DCT coefficient. After $v_{T}$ is obtained, the original signal can be recovered.

### 3.3. Content Recovery by Compressive Sensing

Using the compressive sensing technique, a sparse signal can be retrieved from a relatively small number of measurements. Suppose that x is a column-vector with most elements close to zero and

(17) y=Φ⋅x 

where is Φ a random matrix and y consists of the measurements. Even though the length of y is significantly less than that of x, with the knowledge of the measurements y and the matrix Φ, one can approximately reconstruct the original signal. Several algorithms have been proposed such as orthogonal matching pursuit [30] and gradient projection for sparse reconstruction (GPSR) [29]. Because the reference-values are calculated from the original discrete cosine transform (DCT) coefficients of host speech signal, the reference-values are sparse in DCT domain. Then, view $A^{\left(E,T\right)}$ in (16) as the matrix Φ in (17), and S(1),S(2),...S(M) as a series of measurements of sparse signal. Then, employ the GPSR method in [29] to solve $v_{T}$. Here, the more the available measurements, the more exactly the original coefficients can be retrieved. After getting the vector $v_{T}$, we can calculate the content of tampered frames by inverse DCT.

(18) $$x(n)=\sum_{k=0}^{N-1}\alpha (k)X(k)\cos[\frac{\pi (2n+1)k}{2N}]\begin{array}{ccc} & & 0\leq n\leq N-1 \end{array}\;$$

where

(19) $$\alpha (k)=\left\{ \begin{array}{l} 1/\sqrt{N},\begin{array}{cccc} & & & k=0 \end{array}\\ \sqrt{2/N},\begin{array}{cccc} & & & 1\leq k\leq N-1 \end{array} \end{array}\right.\;$$

where X(n) is the DCT coefficients of the original signal, x(n) is the original speech signal.

## 4. Experimental Results

### 4.1. Subjective Experiment

We choose five sentences from the CASIA-863-speech synthesis database randomly, represented here by the numbers one to five. All speech signals last five seconds. In addition, they were resampled to 16-bit 8-kHz to meet the requirements. The subjective experiment is mainly used to test whether watermarked speech is imperceptible or not.

The subjective difference grade (SDG) is a subjective test method for evaluating the quality of watermarked speech signals. SDG formula is as follows:

(20) $$SDG=Grade_{TS}-Grade_{RS}$$

The $Grade_{TS}$ and $Grade_{RS}$ represent test signal scores and reference signal scores, respectively. The SDG ranges from 0.0 to −4.0 (imperceptible to very annoying as show in Table 1). In the subjective listening test, the original and watermarked speech signals were given to ten participants, who were asked to score the speech signals, the scoring criteria are from 1.0 to 5.0, as shown in Table 1. In addition, Formula (20) was then used to calculate the SDG score. Before the start of the experiment, participants needed to understand the entire experimental process. They were properly trained to effectively assess sound quality. The participants were males and females of different ages (22–25 years) with normal hearing. Table 2 shows the average SDG scores of different watermarked speech signals using the proposed method. From the test results, we observed that the SDG ranges from −0.07 to 0.0 for all watermarked sounds using the proposed method, indicating that original and watermarked speech signals are perceptually indistinguishable.

The ABX method is another technique for subjective quality assessment of watermarked speech. Participants were ten males and females with normal hearing. In the test, the original speech signal ‘A’ and the watermarked speech signal ‘B’ were presented to each participant. A third speech signal ‘X’ was randomly selected from ‘A’ or ‘B’ and presented to the participants. Participants were asked to identify whether ‘X’ is the same as ‘A’ or ‘B’. One identification was considered as one trial and each participant performed five trials. The correction of identification is used to determine if the watermarked speech is perceptible. A detection percentage of 50% indicates that the difference between the original and the watermarked speech is imperceptible. The evaluation results are shown in Table 2. We observed that the correct detection scores ranged from 46 to 54%, indicating that the watermarked sounds was almost imperceptible.

### 4.2. Objective Experiment

The objective experiment is divided into two parts, the first part is used to test the perception of the watermarked speech signal. The second part is used to test the intelligibility of the recovered speech signal.

The objective quality of a watermarked speech signal is measured by SNR. The SNR values of all selected speech signals using the proposed method are shown in Table 2, and we can observe that all the values are above 20 dB, meeting the requirements of International Federation of the Phonographic Industry (IFPI) [31] for audio quality.

Table 3 shows SNR and mean opinion score (MOS) results required for the comparison of the proposed method with several recent methods, which are based on the reported results in [31,32,33,34,35,36]. The MOS method allows the tester to directly compare the original speech with the test speech. The score is from 1.0 to 5.0, similar to the scoring criteria in Table 1. From the comparison of the results, we observed that our method outperforms others in terms of the imperceptibility of the watermarked audio signal. The MOS value of our method is close to 5.0 points, which is higher than other methods.

**Figure 3:**
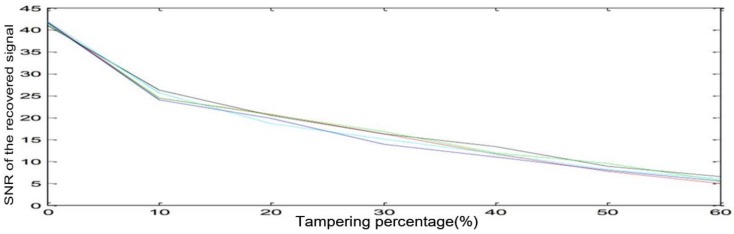
The SNR values of all the recovered speech signal.

The objective quality of a recovered speech signal is also measured by SNR. In the test, the signal of the tamper area is set to mute, and the ratio is 10%, 20%, 30%, 40% 50%, 60% respectively. After tampered area localization, all the parts that are set to mute are found. Subsequently, we extract reference-values from areas which are not tampered, and recover the tampered areas with the reference values. The SNR values of all recovered speech signals using the proposed method are shown in Table 4. In addition, Figure 3 is plotted according to Table 4. The red line represents signal 1, the black line represents signal 2, the green line represents signal 3, the blue-green line represents signal 4, the blue line represents signal 5. According to Figure 3, we can find that the tampering rate of the speech ranged from 10 to 60%. The SNR is almost down from nearly 25 to around 5. The value drops slowly. It can be seen that our algorithm has strong stability when recovering damaged speech signal. The smaller the tampered area, the better the quality of the recovered signal is.

**Figure 4:**
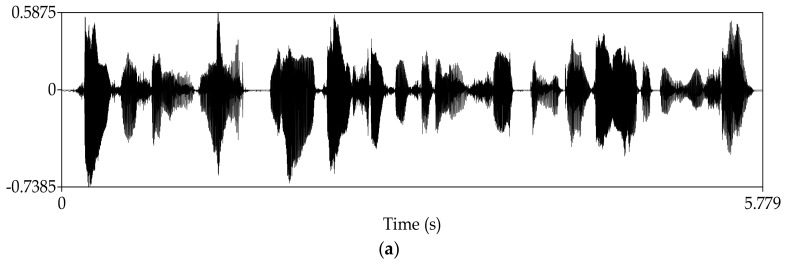

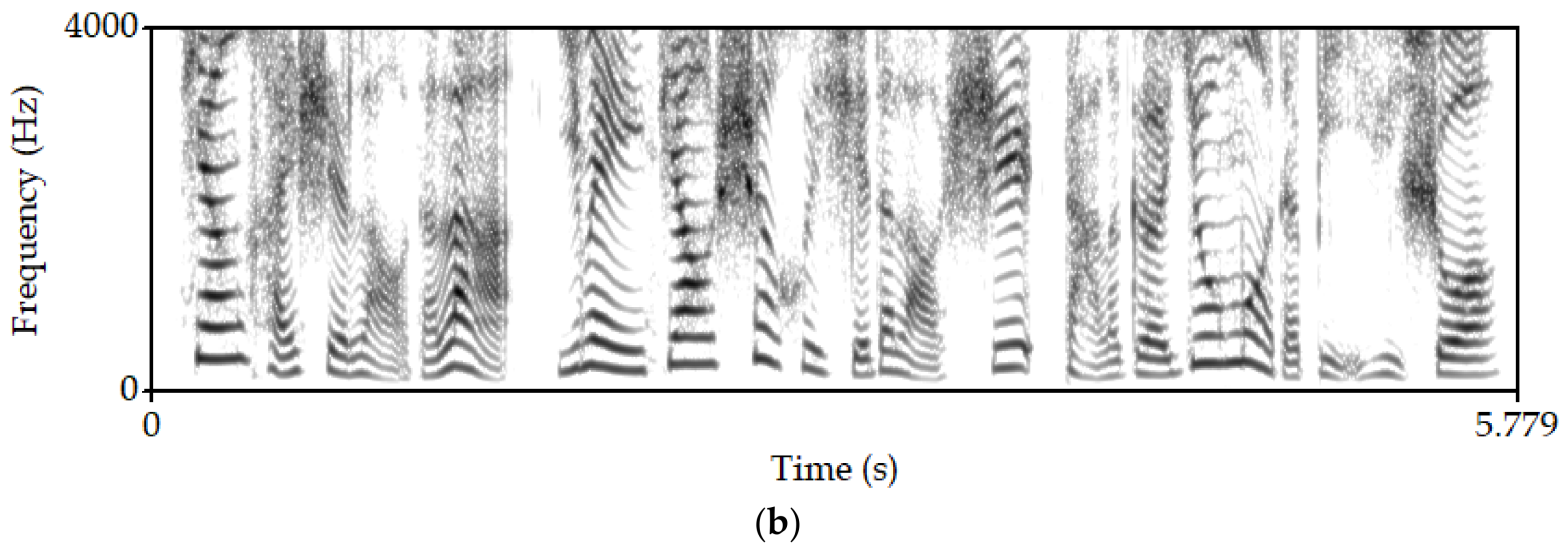
Original speech signal (**a**) Waveform (**b**) Spectrogram.

**Figure 5:**
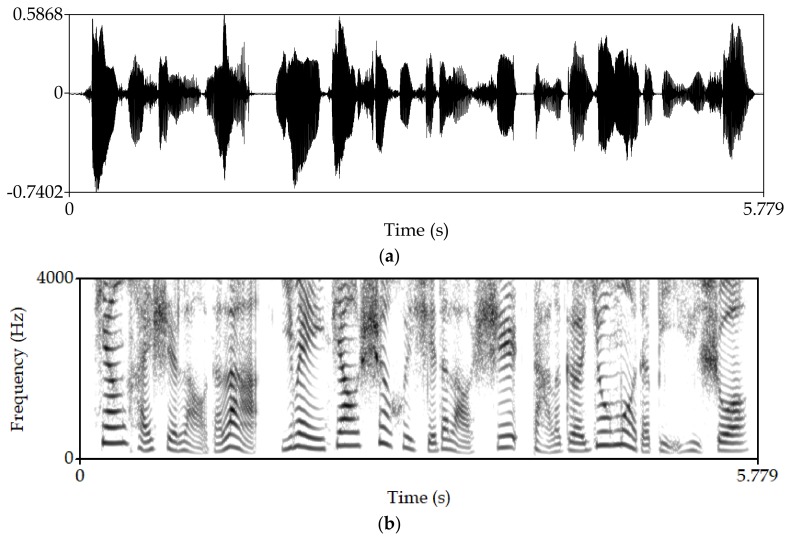
Watermarked speech signal (**a**) Waveform (**b**) Spectrogram.

**Figure 6:**
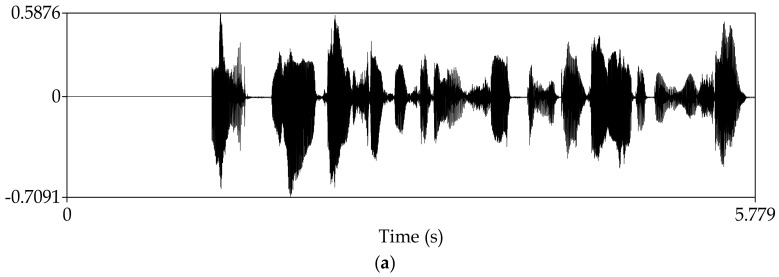

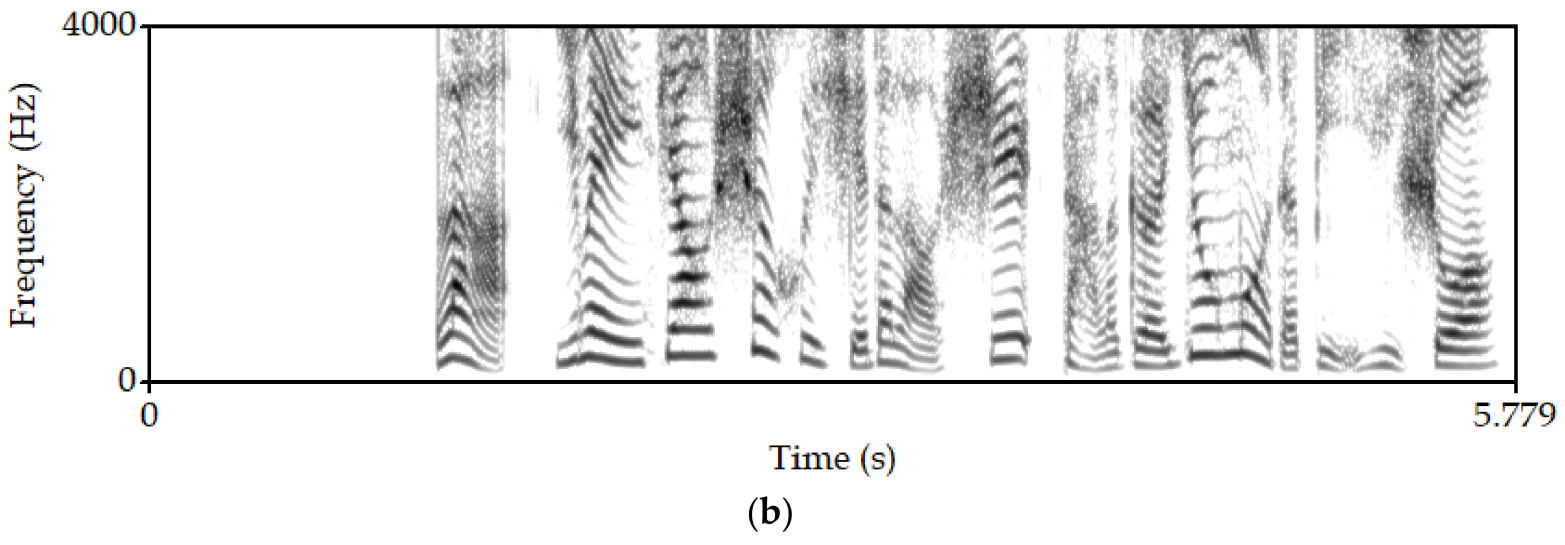
Damaged speech signal, the tampering percentage is 19.5% (**a**) Waveform (**b**) Spectrogram.

**Figure 7:**
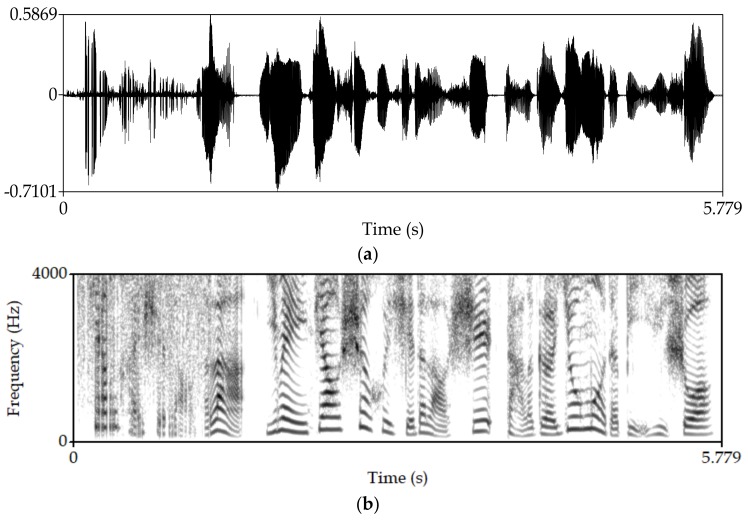
Recovered speech signal, the tampering percentage is 19.5% (**a**) Waveform (**b**) Spectrogram.

Manipulate the speech signal with a tampering percentage of 19.5%. Figure 4 shows the waveform and spectrogram of the original speech signal. Figure 5 shows the waveform and spectrogram of the watermarked speech signal. Figure 6 shows the waveform and spectrogram of the damaged speech signal. Figure 7 shows the waveform and spectrogram of the recovered speech signal. Comparing Figure 4 and Figure 5, it can be found that the original speech signal and the watermarked speech signal are almost identical. This also shows that the watermarked speech signal is imperceptible.

**Figure 8:**
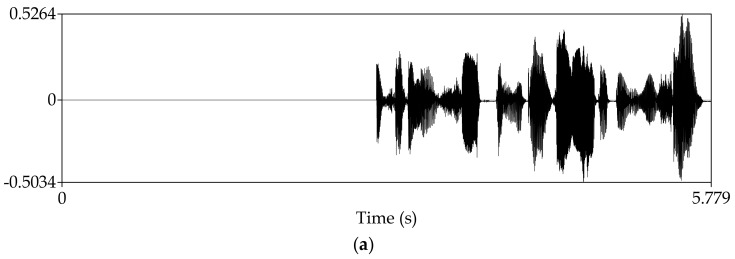

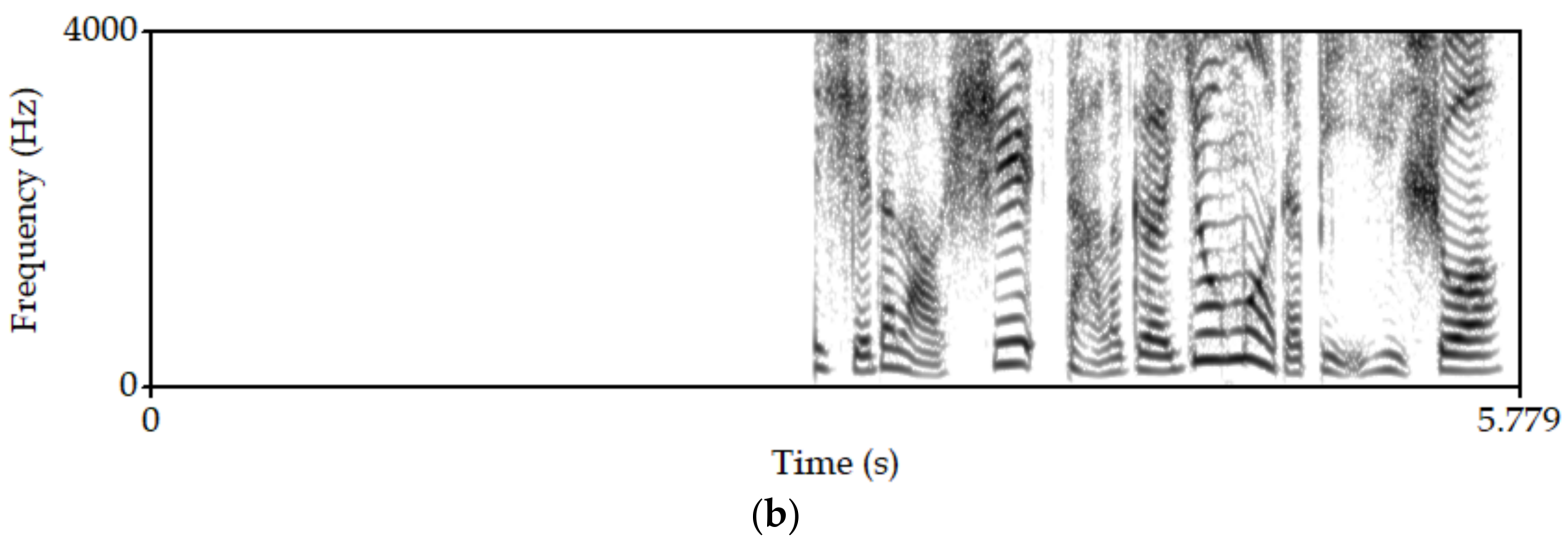
Damaged speech signal, the tampering percentage is 47.6% (**a**) Waveform (**b**) Spectrogram.

**Figure 9:**
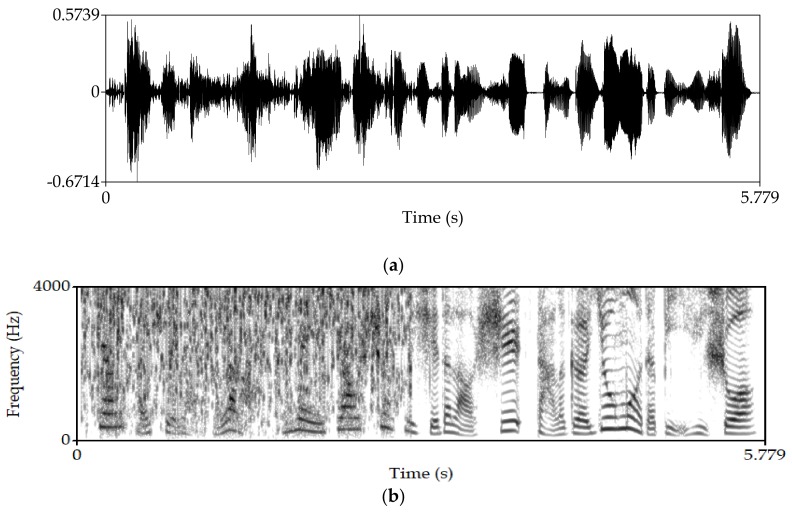
Recovered speech signal, the tampering percentage is 47.6% (**a**) Waveform (**b**) Spectrogram.

Manipulate the speech signal with tampering percentage 47.6%. Almost half of the speech signal is tampered. Figure 8 shows the waveform and spectrogram of the damaged speech signal. Figure 9 shows the waveform and spectrogram of the recovered speech signal. From these figures, it can be seen that when the tampering percentage is as high as almost 50%, our algorithm can still recover the tampered or damaged speech, and the recovery effect is very good.

**Figure 10:**
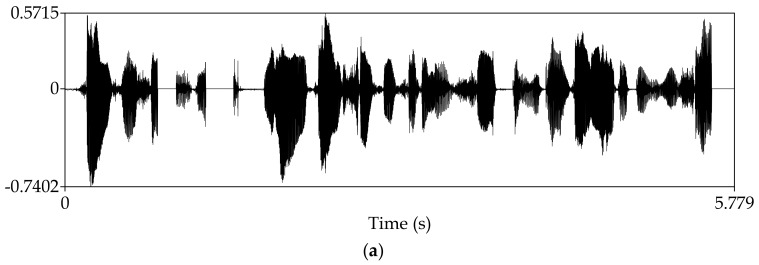

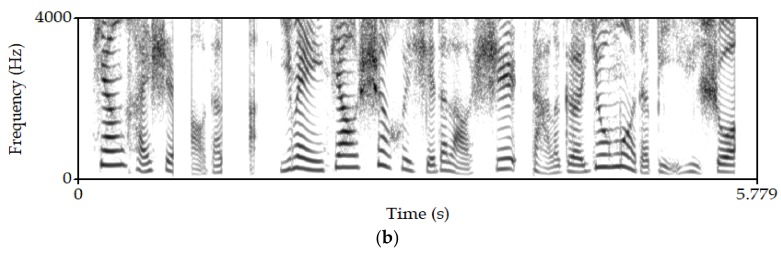
Damaged speech signal, different parts of the signal are tampered separately (**a**) Waveform (**b**) Spectrogram.

**Figure 11:**
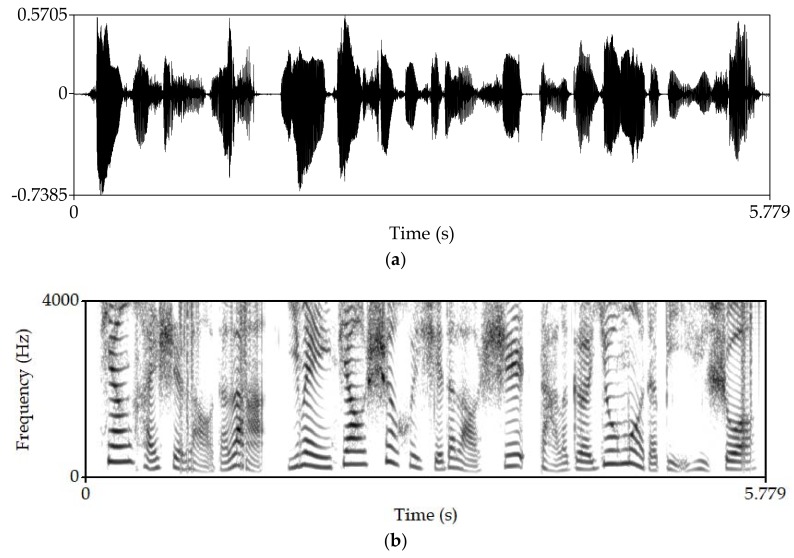
Recovered speech signal, different parts of the signal are tampered separately (**a**) Waveform (**b**) Spectrogram.

Manipulate the speech signal and make different parts of the signal tampered. Figure 10 shows the waveform and spectrogram of the damaged speech signal. Figure 11 shows the waveform and spectrogram of the recovered speech signal. The figures show that the recovery effect is good. In addition, we can see that whether we separately tamper the different parts of the signal or tamper one part of the speech signal, our algorithm can recover the damaged speech signal well.

## 5. Speech Enhancement

We built a deep neural network to continue post-processing of the signal. The overall research process can be roughly divided into three steps: The first step is to build a deep neural network. We use a combination of classical autoencoder and back propagation networks (BP) to build deep neural networks. The second step is to collect data for training and testing. The last step is to train and test the deep neural network model. At this stage, we conduct multiple tests, use the same data set for training under different parameters, and then use the same test set for testing. A comparative analysis of the test results for each experiment, and the results are visually represented by the frequency domain plot, the time domain plot and calculating the signal-noise ratio of the recovered speech signal and the output signal of the networks. 

We build a program for the enhancement to increase the SNR of recovered speech signal. In order to test the influence of the model parameters on the recovery result, we conduct a comparative experiment on the different hidden layer nodes, hidden layers and the iterations of the deep neural network. First of all, we need to build a deep neural network for training with some fixed parameters. After that, we just adjust the corresponding parameters what we need, and use the same training set to train the different network models, and finally use the test program to find the signal-noise ratio, the frequency domain figure and the time domain figure. 

### 5.1. Comparison of the Number of Hidden Layer Nodes

**Figure 12:**
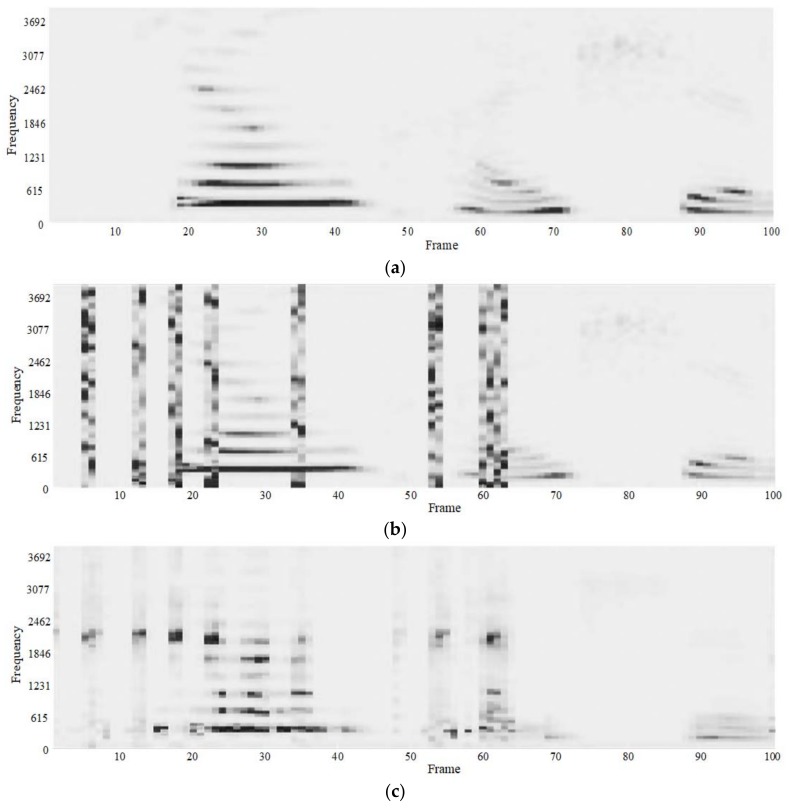

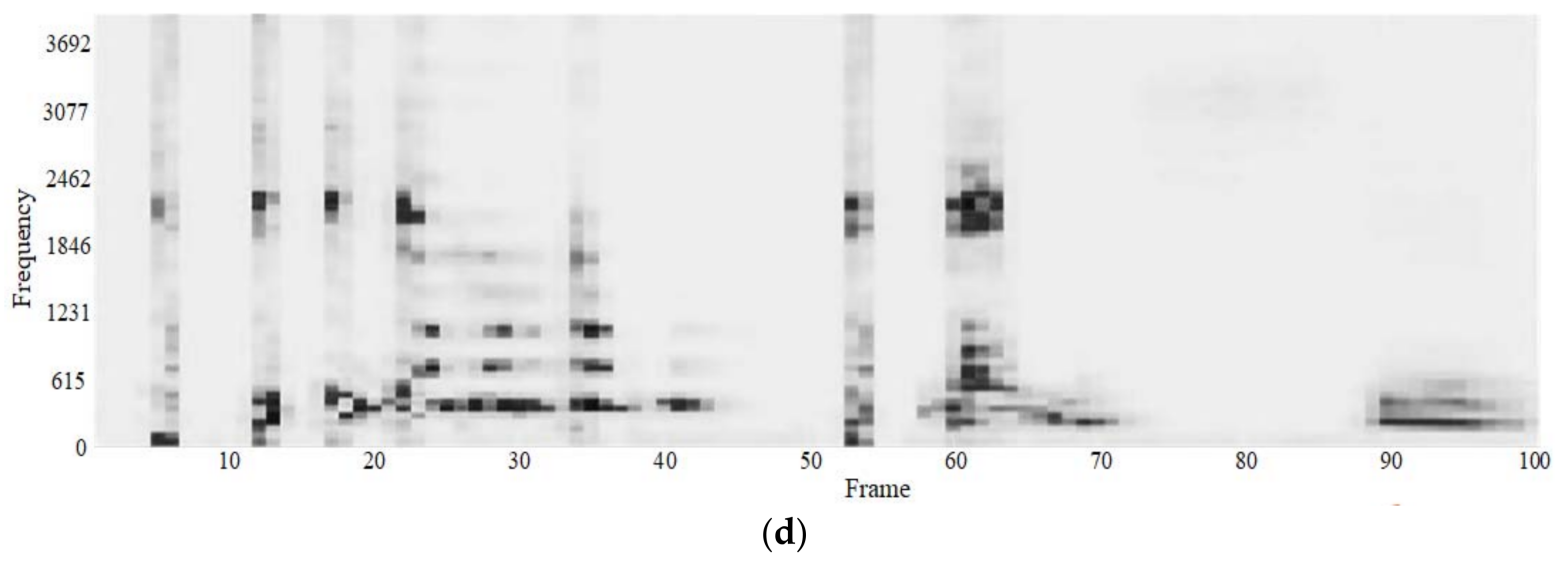
Spectrograms. (**a**) Watermarked speech signal; (**b**) Recovered speech signal without processing; (**c**) Four hidden layers, decrement of nodes; (**d**) Four hidden layers, same number of nodes.

The number of parameters of the seven-layer neural network is higher than that of the four layers. In order to save running time, we first use a four-layer deep neural network for comparison experiments, setting the number of iterations for fine-tuning to 200. The following four frequency domain figures are the experimental results. In Figure 12, (a) is the spectrogram of the watermarked but undamaged speech information, and (b) is the spectrogram of the recovered speech signal. In (c) we set the nodes of four hidden layers to 1000, 500, 100, 30. In (d), the nodes of the four hidden layers are 1000, 1000, 1000, 1000, respectively.

**Figure 13:**
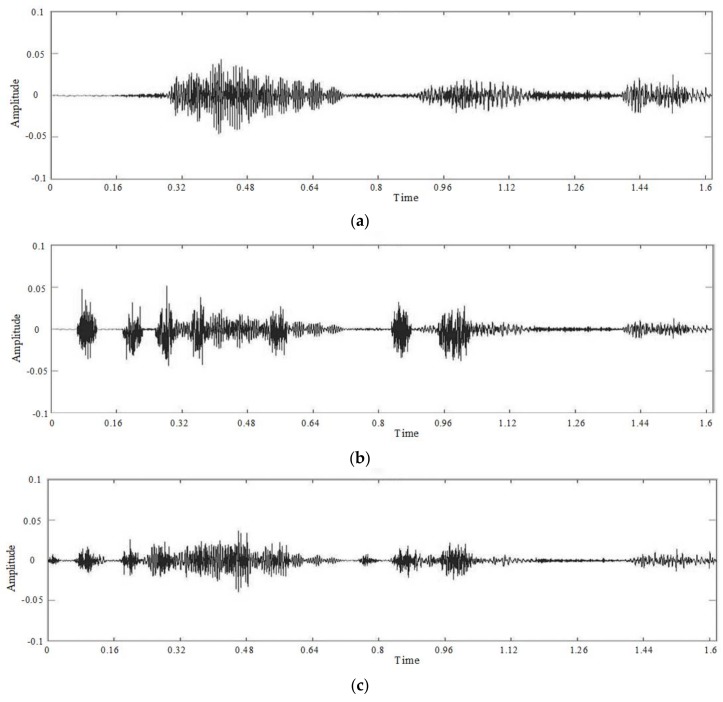

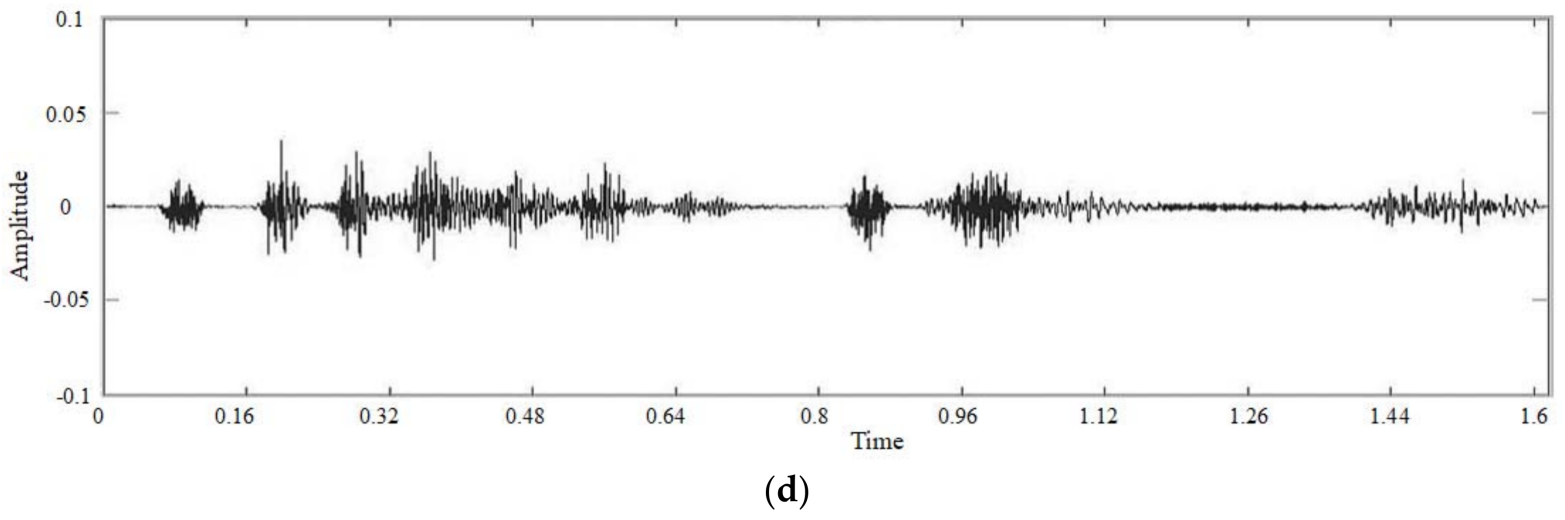
Waveforms. (**a**) Watermarked speech signal; (**b**) Recovered speech signal without processing; (**c**) Four hidden layers, decrement of nodes; (**d**) Four hidden layers, same number of nodes.

From Figure 12, we can see that the original watermarking signal has a good frequency domain figure. In addition, there is still obvious distortion after recovery. We use the network in which the nodes of each hidden layer is decreasing and another network in which each hidden layer has the same number of nodes to enhance it. In addition, the result is greatly improved compared to the watermark recovery effect. We compare the enhanced effect of these two network models in Figure 13.

Table 5 shows the signal-noise ratio values of the recovered speech signal with different numbers of nodes. The SNR of the recovered speech signal without any processing is the lowest. The neural network processing effectively improves the SNR of the signal. The processing results of the network with a decrement in the number of nodes is better, compared to the network with the same nodes for each layer. In addition, we choose the deep neural network model with a decreasing number of hidden layer nodes in the next experiments.

### 5.2. Comparison of the Number of Hidden Layers

**Figure 14:**
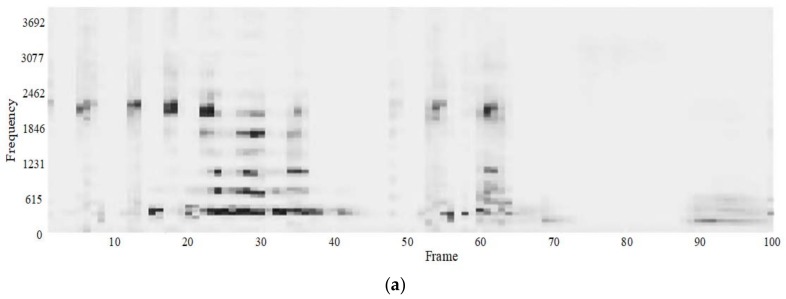

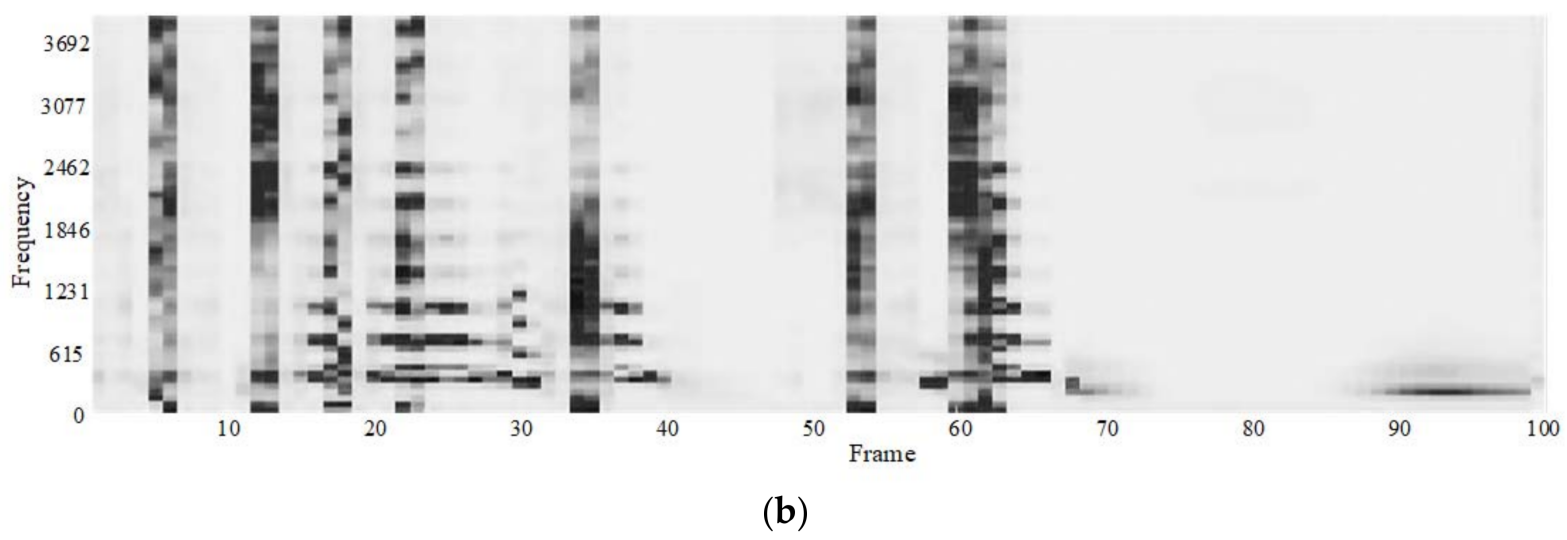
Spectrograms of recovered speech signal using different numbers of hidden layers of DNN (**a**) 4 hidden layers, (**b**) 7 hidden layers.

We construct a deep neural network with four hidden layers and a deep neural network with seven hidden layers. In addition, we set the number of iterations for fine-tuning to 200. The number of hidden layers is different. We use the network in which the nodes of each hidden layer decreases to improve efficiency, and in order to facilitate comparison with the previous model recovery results, the number of the nodes of the four hidden layers is still 1000, 500, 100, and 30, and the number of the nodes of the seven hidden layers is 1000, 750, 500, 250, 100, 75, 50. Figure 14 is a frequency domain figure of the enhanced results of a four-layer network model and a seven-layer network model.

From the two graphs in Figure 14, we can clearly see that the recovery effect of the four-layer deep neural network model is better than the seven-layer network model. The structure of the training data we use is relatively simple, the multi-layered network model produces a certain degree of the over-fitting phenomenon, and the recovery effect is not as good as the network with fewer hidden layers. We can further see the difference from the time domain figure.

**Figure 15:**
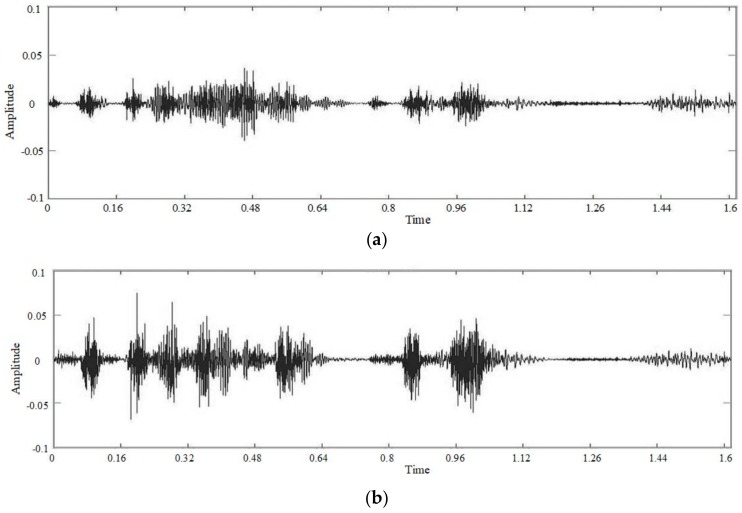
Waveforms of recovered speech signal by using different numbers of hidden layers of DNN (**a**) 4 hidden layers (**b**) 7 hidden layers.

From (a) and (b) in Figure 15, we can clearly see that the signal energy is too high where the signal is distorted after recovery by the seven-layer network model, and does not reduce the apparent noise in the signal, as the four-layer network model does. In addition, compared with the original watermark signal in Figure 13, the speech recovery effect in the whole signal is insufficient. Table 6 shows the signal-noise ratio of the original watermark signal, the output signal of the four-layer network model and the seven-layer network model. The signal-noise ratio of the original watermark signal and the output signal of the seven-layer network model is obviously too low, and the signal is noisy. This result shows that based on the training data set and the test data set we used, the seven-layer deep neural network will produce overfitting phenomenon, and the recovery effect is not as good as a four-layer deep neural network, which has a smaller number of layers.

### 5.3. Comparison of Iterations

**Figure 16:**
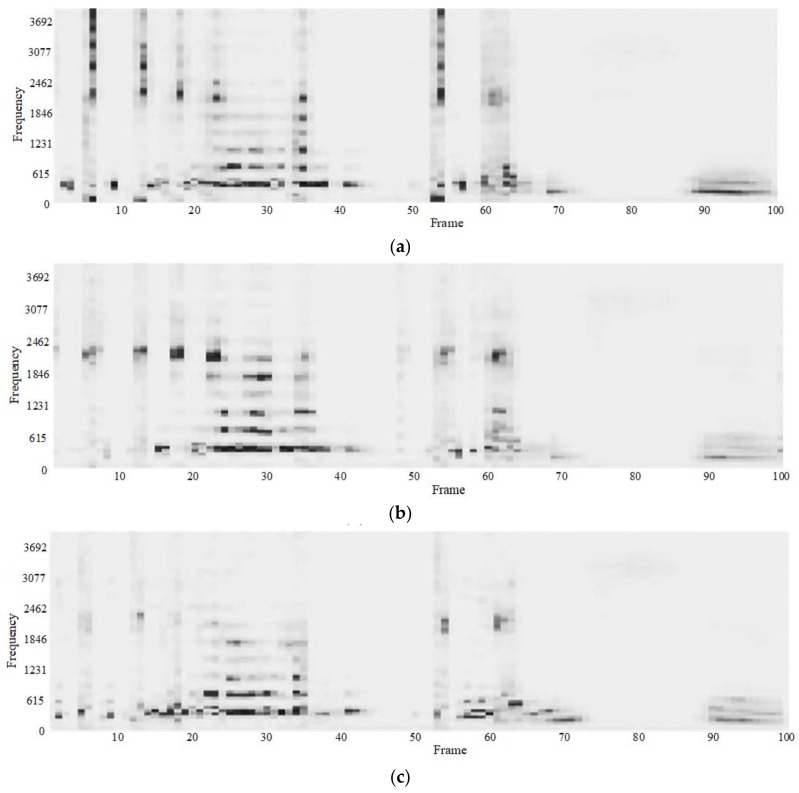
Spectrograms of recovered speech signal by using different numbers of iterations of DNN (**a**) 100 iterations (**b**) 200 iterations(**c**) 500 iterations.

In the comparison experiments of iterative layers, we mainly compare the experimental effect of different numbers of iterations for fine-tuning. In the previous experiment, we used 200 iterations. Now we train and test the network model with 100, 200, and 500 iterations. We also use a 4-layer deep neural network model. The number of hidden layer nodes of the model is 1000, 500, 100, 30. Figure 16 shows the frequency domain figure of the network model for three different numbers of iterations.

From the three spectrograms in Figure 16, we can see that as the number of iterations of the network model increases, the effect of the model on the recovery of the speech signal gradually becomes better. The distortion of the signal is more obvious when enhanced by the network model with 100 iterations. In addition, the distorted parts of the signals enhanced by the models with 200 and 500 iterations are similar to the original watermarking figure in Figure 12.

**Figure 17:**
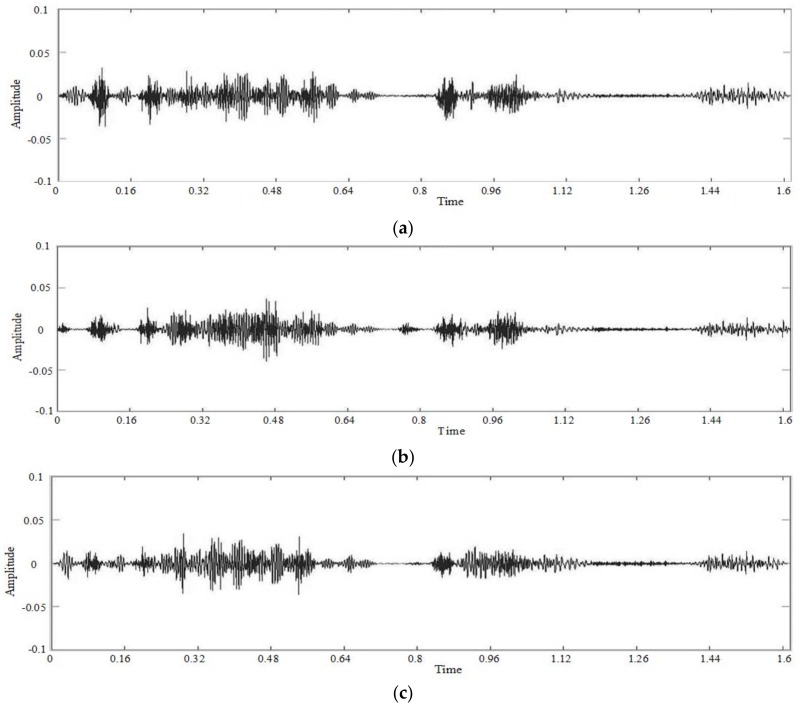
Waveforms of recovered speech signal by using different iterations of DNN (**a**) 100 iterations (**b**) 200 iterations(**c**) 500 iterations.

Figure 17 is a time domain figure of the recovery signal for these three different numbers of iterations of the network model. In the time domain figure, compared with the original watermarking signal in Figure 13, we can clearly see that the recovery effect of the front part and the middle part of the signal increases as the number of network model iterations increases.

Let’s take a look at the signal-noise ratio results. As shown in Table 7, the signal-noise ratio of the output results of the 100-iteration and 200-iteration network models are improved, which proves that the recovery effect is better; however, the signal-noise ratio of the output results of the 500-iteration network model is lower than the 200-iteration model. Due to the fact that when the number of iterations is small, the network model is under-fitting, and the model recovery effect is insufficient, but when the number of iterations is too high, the training model is overfitted, resulting in a decrease in the effect of recovery again. Therefore, selecting a number of iterations between 100 and 500 will make the recovery effect better. The SNR of the recovery signal of 200-iteration model also proves this point. 

## 6. Conclusions

In this paper, we proposed a novel speech watermarking scheme. This method embeds the DCT coefficients of the host speech signal into the LSBs of the host speech signal. When a part of the watermarked signal is tampered with, the watermark data in the reserved area can be extracted. Then we use the compressive sensing technique to retrieve the coefficients in the tampered area due to their sparseness. As a result, the smaller the tampered area, the better quality of the recovered content is. The watermark data information is shared in a frames-group instead of stored in one frame. It has the capacity to make a trade-off between the data waste problem and the tampering coincidence problem. The results indicated that speech could be recovered with reasonable intelligibility when the reference data is sufficient. In addition, the deep neural network can improve the signal-noise ratio of the recovered speech signal. However, there is some noise in the recovered speech signal. In addition, the watermarking in this paper is fragile. It is easy to attack. The fragile watermark lacks robustness. In the future, we will study the post-processing of the recovered speech signal to improve the quality of the recovered speech signal. In addition, we will also focus on semi-fragile watermarking schemes to improve the robustness of the speech signals.

## Figures and Tables

**Table 1:** Subjective difference grade.

Grade	Description	SDG
5.0	Imperceptible	0
4.9~4.0	Imperceptible but not annoying	−0.1~−1.0
3.9~3.0	Slightly annoying	−1.1~−2.0
2.9~2.0	Annoying	−2.1~−3.0
1.9~1.0	Very annoying	−3.1~−4.0

**Table 2:** Subjective and objective evaluation of different watermarked sounds.

Audio Signal	Subjective Evaluation		Objective Evaluation
	SDG	Correct Detection (%)	SNR
1	−0.05	54	41.80
2	0.0	52	40.95
3	−0.07	46	41.32
4	0.0	48	41.93
5	0.0	46	41.68
Average	−0.024	49.2	41.54

**Table 3:** Comparison of SNR and MOS between the proposed method and several recent methods.

Reference	Algorithm	SNR	MOS
[32]	Spread spectrum	28.59	4.46
[33]	STFT-SVD	28.36	4.70
[34]	SVD	27.13	4.60
[31]	DWT-SVD	26.84	4.60
[35]	Frequency masking	12.87	2.93
[36]	TS echo hiding	22.70	4.70
Proposed	DCT-CS	41.54	4.97

**Table 4:** The SNR values of all the recovered speech signal.

	Audio Number	1	2	3	4	5	Average (%)
Tampering Percentage (%)							
0		41.80	40.95	41.32	41.93	41.68	41.54
10		24.47	26.28	24.38	25.60	24.03	24.95
20		20.70	20.42	20.58	18.65	19.77	20.02
30		16.23	16.20	16.80	15.02	13.83	15.62
40		11.67	13.33	11.92	11.79	10.97	11.94
50		7.76	8.90	9.53	8.12	7.91	8.44
60		4.87	6.52	5.60	5.95	5.41	5.67

**Table 5:** The SNR values of the recovered speech signal with different numbers of nodes.

Watermarking—Recovery	Watermarking—Decrement of Nodes	Watermarking—Same Number of Nodes
−0.20249	2.0729	1.3375

**Table 6:** The SNR values of the recovered speech signal with different numbers of hidden layers.

Watermarking—Recovery	Watermarking—4 Layers	Watermarking—7 Layers
−0.20249	2.0729	−2.563

**Table 7:** The SNR values of the recovered speech signal with different numbers of iterations.

Watermarking—Recovery	Watermarking—100 Iterations	Watermarking—200 Iterations	Watermarking—500 Iterations
−0.20249	1.6238	2.0729	1.2703
